# A multi-method pilot exploration of a brief behavioral sleep intervention for school-aged children: feasibility, acceptability, and initial evaluation

**DOI:** 10.3389/frsle.2026.1731331

**Published:** 2026-05-05

**Authors:** Isabella Delores Wright, Gracie Crandall, Scott Baldwin, Kara McRae Duraccio

**Affiliations:** Department of Psychology, Brigham Young University, Provo, UT, United States

**Keywords:** behavioral sleep medicine, brief behavioral intervention, pediatric sleep, pediatrics, sleep

## Abstract

**Objectives:**

Parents often lack knowledge regarding healthy sleep practices, and little is known about whether a brief behavioral intervention emphasizing mastery, personalization, motivation, and modeling can increase parental knowledge of childhood sleep and promote child sleep health outcomes. This multi-method pilot study aimed to (1) assess parental knowledge of healthy child sleep practices, (2) determine the preliminary efficacy of a brief, personalized sleep intervention on parental self-efficacy and child sleep behaviors, and (3) examine the feasibility, acceptability, and parent-perceived impact of the intervention on child sleep health and parental knowledge.

**Methods:**

54 Parent-child dyads in a community sample participated in a 7-day baseline sleep assessment before attending a brief behavioral intervention appointment designed to improve child sleep. Two weeks later, parents completed an additional sleep assessment, participated in a qualitative interview, and reviewed actigraphy data to evaluate changes in child sleep.

**Results:**

Parental knowledge of healthy child sleep practices improved significantly following the intervention (*p* < 0.001, ηp^2^ = 0.245). Self-reported child sleep disturbance (*p* = 0.041, ηp^2^ = 0.084), pediatric insomnia severity (*p* < 0.001, ηp^2^ = 0.213), and parental self-efficacy (*p* = 0.036, ηp^2^ = 0.088) also significantly improved. Objective sleep measures (sleep duration, onset latency) did not change (*p*'s > 0.05), and sleep efficiency declined (*p* = 0.001). Qualitative feedback indicated the intervention was acceptable and feasible.

**Discussion:**

The pilot brief behavioral intervention improved parents' perception of child sleep health, parental self-efficacy, and parental sleep knowledge. Despite limited changes in objective sleep measures, findings from this predominantly subclinical, community-based sample suggest that brief, personalized behavioral interventions may be a feasible and acceptable approach for promoting sleep health and supporting families. Future investigations, including randomized control trials and longer follow-up periods, are warranted.

## Introduction

Current understanding of strategies to enhance sleep in school-aged children (ages 5–12) is an under-researched area of pediatric sleep psychology (Meltzer et al., 2021). The majority of intervention literature focuses on infant/toddler sleep interventions (where parents play a key role in intervention delivery) and adolescent interventions [where adolescents are the intervention target due to their autonomy (Meltzer et al., 2021)]. However, sleep in school-aged children is less examined, perhaps because of the unique developmental changes from toddlerhood to childhood (Mindell and Owens, 2015) and increasing sleep autonomy (Mindell and Owens, 2015). Sleep disturbances are often overlooked by parents who are no longer checking on children regularly (Owens et al., 2000). Unfortunately, school-aged children have not developed sufficient sleep autonomy for direct delivery of sleep interventions (Quach et al., 2011). Indeed, these various factors likely influence the fact that an estimated 41% of school-aged children do not receive adequate sleep (Hawkins and Takeuchi, 2016). Further, parents are rarely educated in healthy sleep practices for school-aged children (Owens and Jones, 2011a). As such, understanding and enhancing sleep in school-aged children is understudied yet clinically essential.

In a recent scoping review of current behavioral treatments for pediatric sleep concerns, Meltzer et al. (2021) were able to identify only nine existing behavioral interventions tested within school-aged children. In comparison, 57 intervention studies exist in younger children and adolescents combined, showcasing the large need for more research within the school-aged population (Meltzer et al., 2021). Behavioral interventions are consistently more effective and longer-lasting than medications across all age groups (Meltzer et al., 2021). However, many behavioral treatments can be time-consuming and burdensome (Dewald-Kaufmann et al., 2019) and, as most pediatricians are not trained in sleep intervention or assessments (Owens and Jones, 2011a), it can be difficult for families to find providers. These difficulties combine to decrease access, especially in school-aged children (Zhou and Owens, 2016).

To decrease the cost and time required, recent behavioral interventions have been designed to be briefer, more targeted, and personalized. In adolescents, this intervention model has been found to be effective in increasing total sleep time, decreasing social jetlag, and increasing perceived sleep quality (Paavonen et al., 2016). For school-aged children who are neuroatypical (primarily autism and ADHD), these brief interventions have been found effective in improving sleep outcomes, signaling that replication for such brief interventions in the general population may be warranted (Pattison et al., 2020; Papadopoulos et al., 2019). As providers are only meeting for 2–3 sessions, they are capable of seeing many more patients compared to a six-to-eight-session protocol (Quach et al., 2011).

To our knowledge, there are very few published interventions that have evaluated brief, personalized, parent-focused behavioral sleep interventions for school-aged children in general (non-neurodivergent) community samples, particularly those aimed at promoting sleep health or addressing subclinical sleep concerns rather than treating formally diagnosed sleep disorders (Busch et al., 2017; Gaskin et al., 2024; Quach et al., 2011). Existing interventions for this age group have primarily been school-based, group-delivered, or focused on treatment of clinically significant sleep problems, rather than individualized parent-delivered sleep health promotion. However, the evidence to date in school-based programs and neurodivergent samples suggests that an intervention model focused on parental education around healthy and unhealthy sleep practices, paired with collaborative and tailored sleep interventions, could be beneficial in promoting sleep health and preventing sleep difficulties for school-aged children. Specifically, many non-sleep-related psychoeducation interventions aim to increase parental self-efficacy for sustained behavioral results (Lawrance and McLeroy, 1986), suggesting that applying this model to brief behavioral sleep interventions is a logical next step.

One proposed avenue for improving sleep among school-aged children includes delivering interventions that promote overall sleep health (Busch et al., 2017). Many children experience suboptimal sleep (due to issues such as poor limit setting, nighttime fears, screen use, poor use of autonomy, etc.) but may not meet thresholds of clinically diagnosed sleep disorders such as insomnia or delayed sleep phase (Busch et al., 2017; Gaskin et al., 2024). Accordingly, an emerging body of literature has examined the efficacy of sleep health promotion interventions, which aim to improve child sleep behaviors in non-clinical samples to address suboptimal sleep and potentially prevent later sleep difficulties (Busch et al., 2017; Gaskin et al., 2024). These interventions have primarily been evaluated in school settings, with varying length and efficacy (Gaskin et al., 2024). Several have also been developed specifically to promote healthy sleep duration, a key component of overall sleep health, though they too vary in length, modality, and efficacy (Bonuck et al., 2022; Cassoff et al., 2013). Notably, there remains a gap in the literature on brief, personalized interventions that target parent concerns or specific gaps in children's sleep health, particularly for school-aged children. Developing such interventions is critical for accessibility, as most children with sleep problems do not require the full CBT-I or broader protocols (e.g., TRANS-C), but rather a brief, tailored approach that can effectively address their singular sleep issue and promote overall sleep health (Busch et al., 2017; Gaskin et al., 2024).

Given the limited knowledge around sleep in school-aged children and the role parents can play in promoting sleep health for school-aged children, we sought to develop a pilot intervention that was brief, personalized, and focused on fostering self-efficacy and behavior change in parents by increasing parental knowledge of sleep health, increasing parent motivation, and presenting evidence-based behavioral practices as personalized and specific to promote overall child sleep health. As such, in this multi-method pilot study, we aimed to first evaluate parental knowledge of healthy child sleep practices in a general community sample, comprised of primarily “healthy” sleepers. We hypothesized that fewer than 50% of parents would know the recommended sleep duration range for their child. Second, we aimed to assess the acceptability of a brief, personalized parental sleep intervention to promote sleep health in school-aged children. We expected qualitative feedback to indicate high acceptability and feasibility (>80% of parents finding the intervention helpful), with themes such as intervention ease, helpfulness, and improvements emerging from interviews. Third, we aimed to examine the pilot intervention's preliminary efficacy on parental sleep knowledge and child sleep health promotion. We hypothesized a 20% increase in correct Sleep Knowledge Survey scores at follow-up, improvement in parent-reported child sleep disturbance, improvement in parent-reported pediatric insomnia symptoms, along with improvements in child sleep duration and sleep onset latency (SOL) based on parent reports and actigraphy. Fourth, we aimed to assess how parents' self-efficacy changed across this intervention, hypothesizing higher self-efficacy scores post-intervention. Finally, we explored potential improvements in secondary sleep and behavioral outcomes, including sleep efficiency and wake after sleep onset (WASO).

## Methods

### Participants

All study procedures were approved by the corresponding author's institutional review board. Parents of and children ages 5–11 were recruited for this pilot study. Flyers were posted physically and electronically at local elementary schools, around the university campus, and throughout the community. Parent/child dyads were included if both a parent and their child of ages 5–11 were interested in participating, and the dyad was able to perform all study procedures; to enhance the generalizability of the study, both mothers and fathers were invited to participate, and participants with comorbid mental health/physical health conditions were included. As a goal of the study was to characterize sleep knowledge in parents without prior clinical sleep care exposure, we excluded participants whose child had a diagnosed sleep disorder or had previously received formal sleep interventions or prescription sleep medications. We did not exclude participants who had taken melatonin, given its widespread use. Additionally, as the interventionists were English speakers only, we excluded participants who did not speak English.

**Figure 1:**
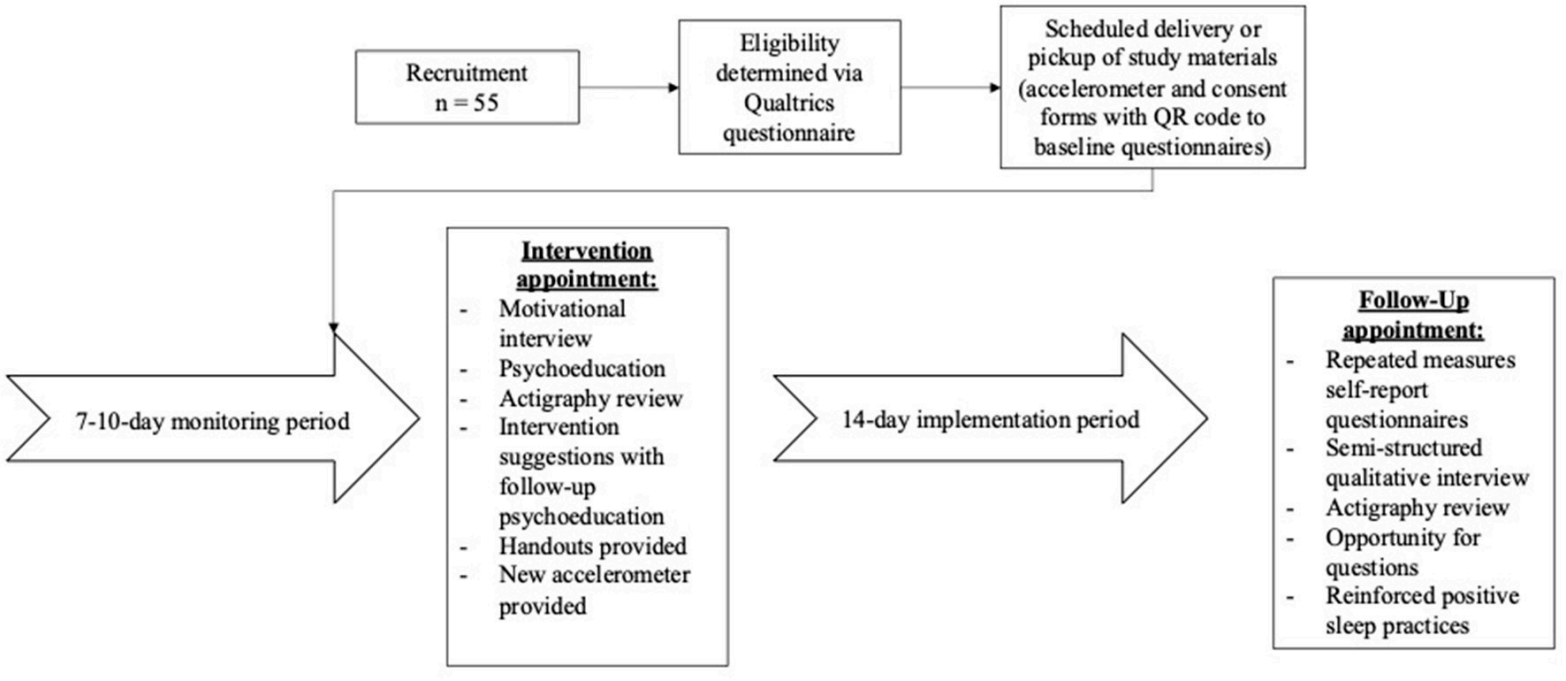
Study protocol.

Interested parents completed an eligibility survey. After determining eligibility, the child and a parent received a package containing: (1) a wrist-worn accelerometer (Actiwatch 2) and instructions on how to have the child wear the wrist-worn accelerometer throughout the study; (2) instructions for completing a Qualtrics questionnaire (with electronic consent and assent forms for the parent and child, and a baseline survey assessing parent-reported child insomnia symptoms, sleep disturbance, perceptions, and parent self-efficacy); and (3) a physical sleep diary to complete each day of the study. Once parents completed the Qualtrics questionnaire, they immediately began a 7–10 monitoring period in which the child wore the wrist accelerometer, and parents completed a daily sleep diary. Following this monitoring period, parents attended the intervention appointment (See Figure 1 for study procedures).

### Visit 1: baseline assessment and intervention

During Visit 1, which included both a baseline assessment review and intervention delivery, parents attended the appointment without the child present. These appointments were held at the university's community clinic. During this appointment, interventionist spent 5–10 min conducting motivational interviewing to assess and enhance the parent's motivation and efficacy for improving their child's sleep. Motivational Interviewing techniques were also used throughout the appointment. The interventionist then reviewed questionnaires and accelerometry data and metrics with the parent. Importantly, actigraphy was used as a supplemental assessment and feedback tool to support personalization and parent engagement, rather than as a required component of the intervention. Identification of sleep problem domains and selection of behavioral recommendations were primarily driven by parent report, standardized measures, and functional analysis. Some examples of problematic behavior that may have been highlighted with or by the parent included: maladaptive sleep-onset associations, bedtime resistance, limit-setting issues, and delayed circadian phase within context of school systems, etc. Following this review of the data and functional analyses, the interventionist worked collaboratively with the parents to identify the top one to three sleep-improvement areas that the parent indicated willingness to implement (see Table 1 for intervention focus). The interventionist screened for symptoms of a possible sleep disorder that would merit further investigation by a medical provider *via* accelerometry, questionnaires, and clinical interview. If organic sleep symptoms seemed to be present, the interventionist referred the participant to their pediatrician for further investigation and identified any other behavioral sleep difficulties that appeared *via* measures and could be improved. Clinical recommendations were then written up and provided to the parent for their three identified sleep goals, with instructions to attempt to complete these recommendations to their best ability for the following 2 weeks. The interventionists in this study were advanced clinical psychology doctoral students who obtained weekly clinical supervision from a licensed pediatric sleep psychologist.

**Table 1:** Prioritization of provided interventions.

Prioritization guide for identifying sleep health optimization targets^*^	Identified sleep problem area	Treatment recommendation
1	Problem areas specifically indicated by parent	
2	Most frequently occurring sleep issues as reported by parent	
3	Co-sleeping or required parental/sibling presence	Phased extinction; fading parental presence; camping out (Lunsford-Avery et al., 2021)
4	Limit setting issues (i.e., leaving bedroom multiple times, leaving room during night, and bedtime resistance)	Bedtime pass (Friman et al., 1999); reward systems, good morning light; differential attending (Lunsford-Avery et al., 2021)
5	Sleep timing issues (i.e., sleep regularity, inappropriate bedtime, and social jetlag)	Setting stable bed and waketimes; setting new bedtimes, phase advancement (Harvey, 2016)
6	Stimulus control	Only using bed for sleep (Jansson-Fröjmark et al., 2024)
7	Sleep restriction	Only using bed for sleep; only getting into the bed when sleepy
8	Nighttime fears (i.e., nightmares, fear of the dark, and generalized anxiety)	Exposures in the dark; worry time; reality checking; coping strategies (deep breathing, progressive muscle relaxation, guided imagery, and meditation; Harvey, 2016)
9	Difficulty waking	RISE-UP routine (Harvey, 2016)
10	Daytime sleepiness	Behavioral activation; nap extinction; emotion regulation strategies
11	Sleep hygiene	Minimal screen use before bed; setting bedroom to be cool; dark; and quiet; fostering a safe sleeping space; avoiding caffeine during the day; avoiding naps too late in day (Lunsford-Avery et al., 2021)

Sleep health optimization targets were identified via functional analysis, self-report, and actigraphy report during first visit, with one to three improvement areas (depending on intensity of treatment recommendation and parent willingness) were selected collaboratively with the parent

### Visit 2: follow-up assessment

Following Visit 1, parents were sent home with a new wrist-accelerometer for their child and a new sleep diary, and parents were encouraged to implement the clinical recommendations for a 2-week period. Following this 2-week implementation period, the parent returned for Visit 2 without the child present. At this appointment, parents completed the questionnaire battery that they completed at the baseline, accelerometers were collected, and parents were given the opportunity to reflect on changes made. All behavioral strategies implemented were praised and reinforced, and the interventionist reviewed the accelerometry and showed progress in sleep throughout the study. Following this feedback, parents had the opportunity to opt into a semi-structured interview about their perceptions of the feasibility, acceptability, and effectiveness of this protocol, which was used as a qualitative element within the study (see **Appendix** for the full interview). Parents were paid $100.00, prorated based on the study tasks completed.

### Measures

#### Children's sleep habits questionnaire

The Children's Sleep Habits Questionnaire (CSHQ) was used in this study to assess current child disturbance, with higher scores reflecting greater sleep disturbance. The CSHQ was developed for use in school-aged children and supplies eight subscales to identify problematic behaviors (Owens et al., 2000). The CSHQ has demonstrated adequate test-retest Pearson correlations ranging from 0.62 to 0.79, depending on the subscale. Further, internal consistency for the CSHQ has shown to be adequate (Cronbach's alpha = 0.68) for global scores in United States populations (Lucas-de la Cruz et al., 2016). Cronbach's alpha for the total score on the CSHQ in this study was 0.85. For this study, global scores of the CSHQ were totaled for analysis and served as a primary outcome of the study to determine intervention effectiveness.

#### Pediatric insomnia severity index

The Pediatric Insomnia Severity Index (PISI) is a 6-item parent-proxy measure designed to screen for pediatric insomnia diagnostic criteria, and which uses DSM-5 criteria for its items (Byars et al., 2016). The PISI was used in the current study to assess for insomnia symptoms in the child as an additional measure of sleep health. In previous research, Cronbach's alpha has ranged from 0.62 to 0.81 (Byars et al., 2016); the Cronbach's alpha for the PISI in the current study was 0.80. The PISI global score served as a primary outcome of the study to determine the preliminary efficacy of the intervention on sleep health.

#### Sleep knowledge questionnaire

We assessed parental knowledge of healthy sleep in young children using the Sleep Knowledge Questionnaire (SKQ; 6). Developed with a convenience sample in primary care settings, the survey assesses child sleep habits, basic parental sleep knowledge, and beliefs about sleep. While the measure lacks formal validation and published reliability values, its content is appropriate for assessing basic knowledge of healthy sleep in school-aged children. Total correct responses were used to evaluate the intervention's preliminary efficacy on parental knowledge. For sample items, see Table 3.

**Table 2:** Sample characteristics at visit 1.

Demographics	Mean ±SD or %
*N*	54
Parent age	43.09 ± 14.33
Child age	9.93 ± 1.75
Parent female (%)	90.7
Child female (%)	42.6
Parent race/ethnicity (%)	
*White*	87.0
*Asian or pacific islander*	9.3
*Multiracial or biracial*	3.7
*Black*	0.0
Child race (%)	
*White*	81.5
*Asian or pacific islander*	7.4
*Multiracial or biracial*	9.3
*Black*	0.0
Income (%)	
*$20,000–$49,000*	5.6
*$50,000–$74,000*	9.3
*$75,000–$99,000*	24.1
*$100,000–$149,000*	38.5
*$150,000 or more*	21.2
*Declined to report*	3.7
Parent education (%)	
*Some college*	9.3
*2-year degree*	5.6
*4-year degree*	51.9
*Professional degree*	18.5
*Doctoral degree*	14.6
Baseline outcome characteristics	
*Parental knowledge of healthy child sleep (PKQ)*	6.9 ± 1.62
Child sleep habits (CHSQ)	
*33% > 41 (N = 18)*	48.00 ± 4.79
*67% < 41 (N = 36)*	37.98 ± 9.48
*Pediatric insomnia severity (PISI)*	14.32 ± 5.91
*Parental self-efficacy (NGSE)*	34.70 ± 3.84
Child baseline sleep characteristics (*N* = 44)^*^	
Sleep time (min)^*^	567.54 ± 54.96
*Sleep efficiency*(%)^*^	81.85 ± 5.81
SOL^*^	21.88 ± 15.67
*WASO* ^*^	59.78 ± 17.53

^*^Measured via actigraph

**Table 3:** Percent of parents answering sleep knowledge questions correctly.

Item	Percent parents who answered correctly (%) pre-intervention	Percent parents who answered correctly (%) post-intervention
Children who do not get enough sleep are more likely to be underweight than overweight (F)	50	81.5
Snoring indicates a child is sleeping well (F)	85.2	90.7
Being under- or overactive can be warning signs that a child is not getting enough sleep (T)	79.6	90.7
Watching TV in the bedroom makes it more difficult for children to fall asleep (T)	98.1	92.6
Children should have the same bedtime and wake time on weekdays and weekends (T)	61.1	92.6
Children only need a bedtime routine if they are having trouble falling asleep (F)	96.3	94.4
Well-rested children do not need an alarm clock to wake up in the morning (T)	50	63.0
The average preschooler needs ~10 h of sleep/24 h (F)	27.8	29.6
Being overweight can increase a child's risk of sleep problems (T)	81.5	90.7
The average school-aged child needs ~8 h of sleep/24 h (F)	66.7	68.5

#### New general self-efficacy scale

To measure parental self-efficacy in supporting their child's sleep, we used the New General Self-Efficacy Scale (NGSE). The NGSE has a test-retest reliability of *r* = 0.74, and an internal consistency of 0.87 (Baran and Janowski, 2023; Chen and Gully, 2001). The NGSE had a Cronbach's alpha of 0.86 in the current study. Global scores were used as an outcome for the preliminary efficacy of the intervention analyses. This measure was chosen because there are currently no validated measures of parental sleep self-efficacy. The NGSE has been used in pediatric populations to measure parental self-efficacy related to health behaviors and was chosen to align with the intervention's goal of boosting self-efficacy (Ystrom et al., 2008). Additionally, the NGSE has been shown to correlate significantly with measures of broader parental self-efficacy (Sevigny and Loutzenhiser, 2010).

### Sleep outcomes

To obtain total sleep metrics from wrist-worn ActiWatch recordings, data was downloaded in 60-second epochs using ActiWatch software. Parents completed sleep diaries were used to mark the start and stop of the rest interval. When sleep diaries were missing, rest intervals were defined using sustained activity cessation and resuming, and light levels decreasing and increasing, as determined from actigraphy, following standard scoring conventions (Chow et al., 2016; Meltzer et al., 2012). Two independent raters scored all rest intervals, and discrepancies were resolved by averaging rest periods. A sleep report was generated using Sadeh's (2011) algorithm, including SOL, sleep duration, sleep efficiency, and WASO. Sleep duration and SOL were primary outcomes, while sleep efficiency and WASO were exploratory outcomes.

### Power analysis

An *a priori* power analysis was used to determine optimal sample size for this study. Using G^*^Power 3.1.9.7, to have at least 80% power to detect a large effect size of 0.80 (Blake et al., 2017) for a reduction in sleep problems as measured by self-report and accelerometry using paired-sample *t*-tests with a 0.05 two-sided significance level, we would need 23 participants. However, to add robustness to the dataset, and to increase power to detect medium effects, the target number of participants for this study was 55 to account for data collection errors (i.e., participants discontinuing the study, accelerometer malfunctioning). Previous pilot studies have used comparable sample sizes as this study (Loring et al., 2016; Papadopoulos et al., 2015; Sciberras et al., 2011).

### Statistical analyses

Before conducting statistical analyses, we ensured that certain statistical assumptions were met. Specifically, all paired observations were independent, and the distribution of the variance of the primary variables was normally distributed. We tested normality using Shapiro-Wilk tests (*p*'s < 0.05). For all primary analyses, the *p*-value was set at *p* < 0.05; for all exploratory analyses, the *p*-value was set at *p* < 0.01. While we reported on statistical significance, the focus was on effect sizes; we interpreted any finding with a medium effect size (partial eta-squared > 0.06). Any missingness was handled with listwise deletion.

#### Preliminary efficacy of intervention

We used a multivariate analysis of covariance (MANCOVA) to examine how parental self-efficacy, parental knowledge, child mental and behavioral functioning, and parent-reported child sleep health (insomnia severity, child sleep habits) were altered pre- to post-intervention. MANCOVAs were selected to allow for the examination of multiple independent variables while controlling for relevant covariates; covariates that were tested using Pearson Correlations (see Supplementary Table 2) and *T*-Tests (for dichotomous variables) included biological sex, age, race, income, and parental education level. When testing for covariates, child age was significantly correlated with parent self-efficacy, and parent gender was also significantly associated with parent self-efficacy and were thus used as covariates in the model. Further, income was significantly correlated with child sleep habits and was used as a covariate in the model. Parent race emerged as significantly associated with child behavioral health and was included as a covariate in the model.

For accelerometry-measured sleep outcomes (SOL, sleep duration, sleep efficiency, and WASO), we used an applied longitudinal data analysis approach, in which we used all days of accelerometry pre- and post-fit to a multi-level model that allowed us to determine the shape of change from baseline to intervention appointment, and from intervention appointment to follow-up. Further, for all accelerometry-measured outcomes, we ran a model fit analysis to determine if a linear, cubic, or quadratic model was most appropriate for analyses. This model compared a linear to cubic model, and a linear to quadratic model. There were no significant differences between the linear and cubic models or the linear and quadratic models (*p*'s > 0.05) and therefore we concluded a linear model to be the best fit (see Supplementary Table 1).

#### Feasibility and acceptability: qualitative analysis

Interview data was analyzed using both inductive (i.e., allowing themes to emerge naturally) and deductive (i.e., looking for predetermined themes) methodologies. Following recommended procedures by Creswell et al., NVivo 14 software was used to transcribe intervention sessions (Creswell and Poth, 2016). Prior to coding, several themes were hypothesized using deductive methods, including themes around the domains of acceptability, feasibility, and preliminary efficacy. Specifically, we theorized that within the acceptability domain, participants would report finding the overall intervention helpful and finding specific evidence-based practices helpful for their child. We theorized a desire for additional content. Within the feasibility domain, we theorized that ease, convenience, and difficulty integrating the intervention into everyday routine would emerge as possible themes. Lastly, we theorized that sleep knowledge improvement, applicable knowledge (e.g., sleep knowledge would help more than just their child), child's sleep improvement, and increased self-efficacy would emerge as potential themes (see Table 5).

**Table 4:** Self-report and accelerometry-measured outcomes.

Self-report outcome	M(SD) pre-intervention	M(SD) post-intervention	Coefficient	*F*	*p*-value	Partial eta squared
Parental self-efficacy	34.70 (3.84)	36.14 (3.41)		4.633	0.036^*^	**0.088**
Child sleep disturbance	37.98 (9.48)	35.79 (7.77)		4.425	0.041^*^	**0.084**
Pediatric insomnia severity	14.32 (5.91)	12.25 (5.49)		13.492	< 0.001^**^	**0.213**
Parental knowledge of healthy child sleep	6.9 (1.62)	7.9 (1.09)		16.575	< 0.001^**^	**0.245**
*Accelerometry outcome*	*M(SD) pre-intervention*	*M(SD) post-intervention*	*Coefficient*	*z*	*p-value*	*95% confidence intervals*
Sleep duration	563.67 (81.61)	555.43 (59.90)	−2.51	−0.35	0.727	[−1.481, 0.585]
Sleep efficiency	81.84 (7.61)	82.72 (6.58)	−2.19	3.55	< 0.001^**^	[0.981, 3.398]
SOL	24.76 (25.36)	25.82 (24.14)	−0.43	−0.17	0.868	[−5.625, 4.748]
WASO	564.78 (17.53)	57.98 (20.34)	−3.48	−1.68	0.092	[−7.550, 0.570]

^*^p > 0.05;

^**^p > 0.01

Bolded values = moderate to large effect sizes

The descriptive statistics show a simple average increase from pre-intervention to a full 14-day post-intervention appointment in sleep efficiency, while the mixed-effects model reveals that efficiency decreases over time for individuals, which is a more dynamic insight into the data

**Table 5:** Qualitative findings. *Inductive and deductive acceptability, feasibility domains*.

Domain I: acceptability themes	Description of theme	% of sample endorsed (*N* = 19)	Example quotation
ActiWatch Unhelpful	Parent did not find the actiwatch to be helpful	22.22%	“I'm not sure how helpful the watch actually was, because … the 1st week he was kind of uncomfortable about it”
Cash incentive	Parent indicated that payment for participation helped with adherence to protocol.	22.22%	“Yeah, I guess it was easier because we had an incentive. So maybe I'll have to come up with [an] incentive to get it to work”
Child excitement	Parent indicated child excited to participate in the study	33.33%	“I don't know, he was really excited to do this study. That probably helped him going to bed more … He was like, ‘Oh, I'm going to do a sleep study like this … I'm Going to sleep!”'
Did not try intervention	Parent reported not engaging in provided treatment recommendation	27.78%	“I wanted to do the alarm thing, but she's such a sensory person …I'm not going to do that.”
Enjoyed personalized specific interventions	Parent reported enjoying the personalized and specific nature of treatment recommendations provided	16.67%	“I liked that it was tailored to my child and my family experience instead of being like, ‘… do these five … and just a generalized thing for everybody'. But it was like, no, for this particular child, this is probably going to be most beneficial. And it's also a thing you can actually do. So that for me was super helpful.”
Enjoyed sleep diary tracking	Parent reported that filling out the sleep diary was helpful.	22.22%	“It was helpful to write down the time she went to sleep when she was waking up, so I could actually see how long she was sleeping.”
Enjoyed the ActiWatch	Parent reported that they enjoyed wearing the actiwatch as part of the intervention.	44.44%	“She was excited to be wearing the watch. She was kind of intrigued. So I think that is good.”
Expert opinion is motivating for child	Parent reported that having the interventionist provide psychoeducation and treatment helped motivate child to engage in treatment recommendations.	44.44%	“There have been times where I've just taken electronics completely away. Right now he has access to them. And …' he's been putting them away, like the other night, because of the study. And so t'at's been t'at's been a helpful piece of it. I think sometimes just having …an outside person can be helpful.”
Helpful^*^	Parent reported the intervention overall was helpful.	94.44%	“I thought it was great.” “I think it's always helpful to have new information and to and to implement it.”
Implementing a routine	Parent reported that the study protocol itself provided a routine that helped with sleep health.	27.78%	“I think being part of the study, even putting on the accelerometer, was part of his routine. It kind of got him in the mindset of going to bed.” “You know, sometimes in the past, he's resisted going to bed. He wants to stay up. He wants to read and I think he put down [his books] and went to bed because he was doing this thing. Yeah. So again, I understand it's probably more likely to be routine.”
Intervention ineffective	Parent reported the provided treatment recommendation(s) was not effective for their child.	22.22%	“The relaxation thing we tried. Twice. He said it didn't help.” “The night routine. Sometimes it works and sometimes it doesn't.”
Knowledge was helpful^*^	Parent reported the provided psychoeducation was helpful.	83.33%	“It's been really informative, it's been really helpful to find out [things we didn't know about her sleep before]”
Liked seeing watch results	Parent reported they found walking through the actigraphy report with the interventionist to be helpful.	11.11%	“Yeah, it was super, super helpful for me to, to find something that was actually implementable that I can actually do… and I could see the results. That for me is really helpful to be able to not just be like, in theory, this thing should work, but here is what it *actually* looks like. Here's the real data that shows that it did work and this is how it works, because that to me-… I feel armed right? And I feel like I have a leg to stand on when I'm telling my son, ‘you can't have a light on at night, it's for your own good'. And I know only because I saw the data … so that that was really super helpful to me.
No surprises	Parent reported that there were not aspects of the study experience that I them.	11.11%	“No, I don't think there's any surprises.”
Nothing unhelpful	Parent reported that they didn't find anything in the study experience to be unhelpful.	66.67%	“I didn't really find anything unhelpful.”
Specific treatment recommendations were helpful^*^	Parent reported personalized treatment recommendations to be helpful for their child.	88.89%	“I feel like the getting her up to go to the bathroom. Seems like it really helped as far as the bedwetting” “I wasn't sure if she would actually do [the bedtime pass]. I think that actually surprised me that it that it worked as well as it did”
Study not helpful or neutral	Parent reported ambivalence about the efficacy of treatment recommendations.	38.89%	“And whether it's completely solving it is the thing that's still just unknown.”
Study was beneficial to the parent	Parent reported they found the study helpful for themselves.	38.89%	“It was good for me”
**Domain II: feasibility themes**	**Description of theme**	**% of sample endorsed (N** = **19)**	**Quote**
Convenience	Parent indicated the study was convenient to participate in.	27.78%	“No, I felt the study was fairly reasonable in its design and requirements.”
Difficulty filling out sleep diary	Parent reported difficulty filling out the sleep diary.	33.33%	“it was it is hard to know exactly when they fell asleep” “it was work for me to remember to do the log. I think if I had an app or something that would have been super helpful. Just click, click, click through it, you know.”
Difficulty with specific intervention	Parent reported the specific treatment recommendations provided were difficult to implement.	50.00%	“I think it stressful to try to [move our bed time], but I think the results were positive.”
Ease^*^	Parent reported the study protocol was easy to implement.	88.89%	“This was easy.” “It wasn't difficult.”
Navigating life^*^	Parent reported external factors that made implementing the intervention difficult.	83.33%	“I think if it had been any other week, it would have been a lot easier. Our car broke down and then it was spring break so [my son] was at his grandma's house so this all made it a little more difficult.”
**Domain III: preliminary efficacy themes**	**Description of theme**	**% of sample endorsed (N** = **19)**	**Quote**
Child didn't have baseline sleep difficulties	Parent reported that child didn't have baseline sleep difficulties, so they didn't expect any significant sleep changes throughout the intervention period	22.22%	“I didn't go into this thinking that he had sleep problems. And that seems to have been borne out by the data. He sleeps very well … It's nice to see that he didn't seem to have problems wearing the device, which again, was expected.”
Child motivation increased	Parent reported that their child's motivation for healthy sleep increased during the intervention period	55.56%	“I think it's very important for motivation. She had a better understanding and she felt more motivated”
Continuing intervention	Parent reported wanting to continue to implement treatment recommendations past period of study participation.	33.33%	“I tried to do a few of them, and I want her to keep doing a lot of them.”
Daytime behavior improved	Parent reported noticing improvement in child's daytime behavior throughout the study period.	22.22%	“I feel like I saw less negative behavior.”
Increased awareness	Parent reported gaining increased awareness of their child's sleep through study protocol.	77.78%	“[There are a lot] of the things I knew before, but definitely I'm more aware of [her sleep] and more conscious to make sure all of my kids are getting the sleep that they need.”
Increased conversation	Parent reported engaging in more conversation at home regarding their child's sleep.	22.22%	“I think it's always a good idea to be thoughtful about your child's sleep. And even just participating in the study, we were talking about why sleep is important. And so those are all good things to talk about with your kids”
Increased knowledge^*^	Parent reported increased knowledge about sleep health.	94.44%	“Therapist: do you feel like you have more knowledge around sleep? Participant: Yes. A little more. A little bit more.”
Increased patience	Parent reported having increased patience with their child due to understanding sleep better.	11.11%	“Yeah, maybe I can give them a little bit more grace. And that just helped me have understanding better of what's going on and realizing, ‘Oh, there's a lot going on that I'm totally not aware of'.”
Knowledge did not increase	Parent reported they did not gain increased knowledge through study protocol.	27.78%	When probed on if knowledge increased: “Not very much. The particulars of his sleep a little bit, but in general, I don't think so.”
Knowledge is applicable to others^*^	Parent reported the knowledge they gained in the study could be applied to others.	27.78%	“I was maybe a little bit more educated on that it can [peers], which I hadn't really thought about.”
Parent doesn't understand child's sleep issue	Parent reported not understanding why their child struggles in sleep domains despite participation in the study	27.78%	“I still don't understand why she struggles, you know, falling asleep.”
Parent motivation increased	Parent reported feeling more motivated to improve their child's sleep health	5.56%	“I guess I may be more motivated.”
Parent self-efficacy Increased^*^	Parent reported feeling more equipped to help their child's sleep in the future	94.44%	Therapist: and do you feel like if different sleep problems pop up for the child that participated in our study? Do you feel better equipped to help with their sleep at all in the future?” Participant: “Yeah.”
Sleep did not improve	Parent reported their child's sleep did not improve through study protocol.	22.22%	“I think that it's I think it's always helpful to have new information and to and to implement it. And on whether it's completely solving it is the thing that's still just unknown.”
Sleep improved^*^	Parent reported seeing improvement in their child's sleep.	72.22%	“I did see improvement in sleep in shortening the fall asleep time.” “Our first interview [you told me an important thing] is to have kids sleep on [their] own. That is helpful. I didn't know that. But I implemented that and I think it really helped”
Sleep improved due to placebo	Parent reported child's sleep improved to perceived placebo effect.	5.56%	“He's been sleeping good from when we first reached out … he's doing better. Maybe that's a little early placebo thing.”
Sleep prioritization increased	Parent reported prioritizing their child's sleep more than at baseline.	33.33%	“I am prioritizing sleep more, as a health need. As important as if she wasn't eating enough, you know, sleeping is [as important]. It's just good to have that more at the forefront”
Specific intervention improved sleep	Parent reported specific treatment recommendation improved their child's sleep.	27.78%	“I think [the RISE-UP routine] did help. I mean, just the fact that a couple mornings he was ready to wake up and just got right out of bed.”

Themes marked with an asterisk represent deductive codes derived from pre-specified interview domains. All other themes were generated inductively from participant narratives during analysis.

A codebook was created prior to coding the transcripts for all deductive (pre-hypothesized) themes. Transcripts were then double-coded by two separate coders. For exploratory purposes, using inductive methodology, common themes across interviews that were not pre-determined emerged and were added to the codebook (e.g., enjoying the ActiWatch, difficulty with the sleep diary, continuing with the intervention; see Table 5 for a comprehensive list), and all transcripts were re-coded by two separate coders to identify if added inductive themes emerged. All themes had an inter-rater reliability equal to or higher than κ ≥ 0.80.

## Results

### Participants

**Figure 2:**
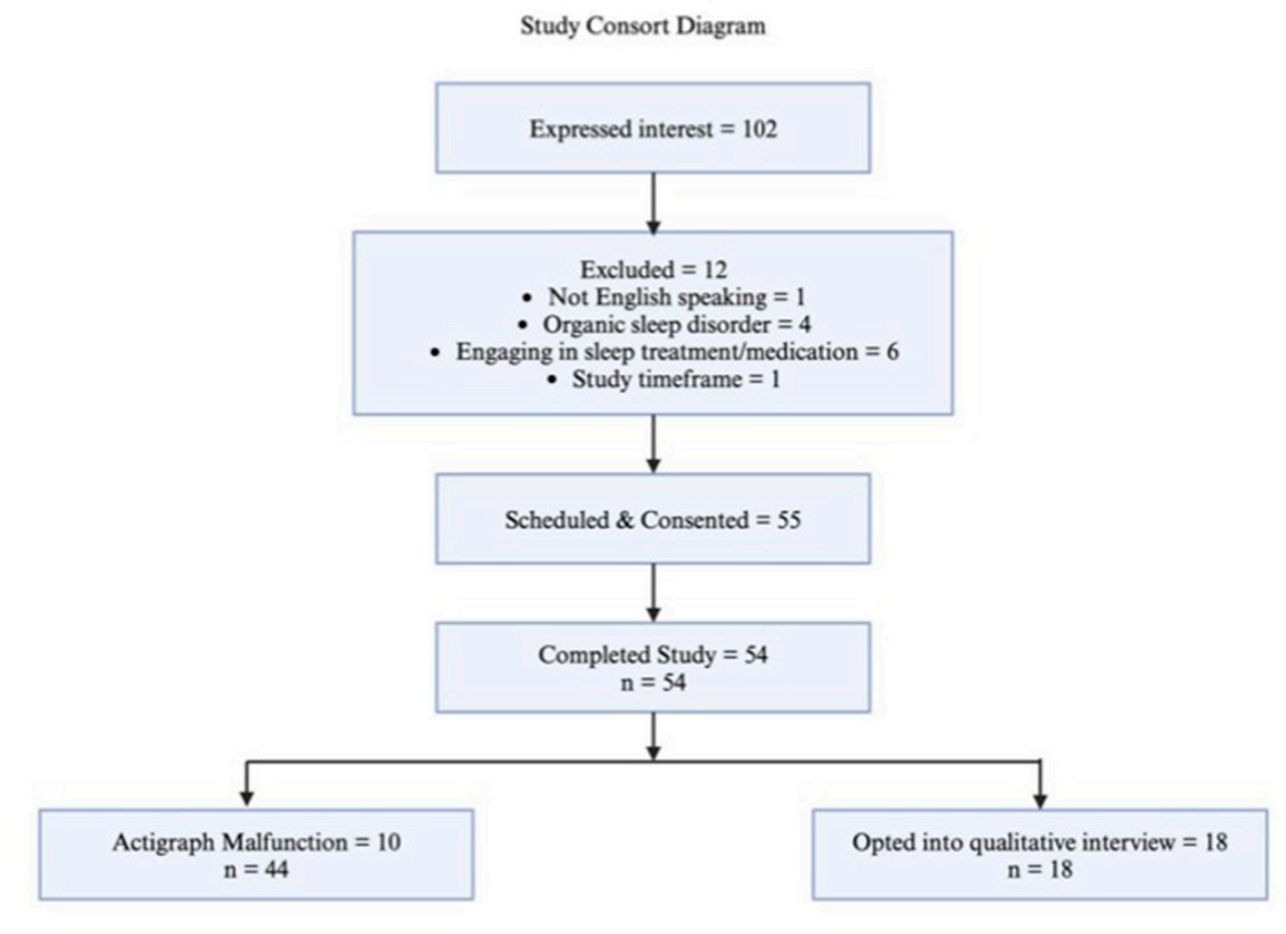
Study consort diagram.

55 participants provided informed consent, and 54 participants completed the study protocol (see Table 2; See Figure 2). Of the 54 participants, we had complete self-report data, resulting in a sample size of 54 for the primary hypotheses (mean child age = 9.9 years old, 87% white, 90.7% of parents identified as female, 42.6% of parents reporting their child to be female; 33% of participants presented with clinically significant parent-reported sleep disturbance; see Table 2). However, due to ActiWatch malfunction or adherence difficulty, we collected accelerometry data on 44 participants, resulting in a sample of 44 participants for actigraph-measured outcomes. There were no significant differences in demographic or baseline sleep characteristics between the final sample and the 44 in which we had accelerometry for (*p*'s < 0.05). Of the 54 participants who participated in the study, 18 elected to participate in a semi-structured qualitative interview regarding their experience in the study.

### Primary outcomes

#### Parental knowledge of healthy sleep in children

The study assessed parents' baseline knowledge regarding children's sleep using the Sleep Knowledge Survey which asks parents to correctly answer 10 true/false questions about childhood sleep. 98.1% of parents successfully identified that having a TV in the child's bedroom would be problematic for sleep (See Table 3). A significant portion also correctly recognized that children need a bedtime routine (96.3%) and that being overweight can increase a child's risk of sleep problems (81.5%). Conversely, only 27.8% of parents correctly identified the average sleep requirement range for children, and just 50% of the parents knew that well-rested children do not need an alarm clock to wake up in the morning (See Table 3). Specifically, 7.4% of the sample answered all 10 questions correctly, while another 7.4% answered only four questions correctly, indicating a broad range in sleep knowledge among parents (see Table 3). We observed that parental knowledge of healthy sleep in children improved significantly from pre- to post-intervention, with large effect (*p* = < 0.001, ηp^2^ = 0.245; see Table 4), with a mean number of correct answers increasing from 6.9 to 7.9, indicating the intervention was effective in increasing parental knowledge of healthy sleep in children.

#### Child sleep disturbance (CSHQ)

Child sleep disturbance global scores improved significantly from pre- to post-intervention with medium effect (*p* = 0.041, ηp^2^ = 0.084; See Table 4), indicating the intervention was effective in improving sleep disturbance, even when covarying for income. The change in child sleep habits and income interaction was not significant (*p* = 0.632 ηp^2^ = 0.005).

#### Pediatric insomnia severity

Pediatric insomnia severity global scores were significantly improved from pre- to post-intervention with a large effect (*p* = < 0.001, ηp^2^ = 0.245; see Table 4), with mean scores increasing from 14.32 to 12.25, indicating the intervention is effective in reducing pediatric insomnia severity.

#### Parent self-efficacy

When covarying for child age and parent gender, parental self-efficacy improved significantly from pre- to post-intervention, with moderate effect (*p* = 0.36, ηp^2^ = 0.088; see Table 4), with mean scores increasing from 34.70 to 36.14. The pre- and post- mean differences in the change in self-efficacy and child age interaction also emerged as significant, with moderate effect (*p* = 0.024, ηp^2^ = 0.102), indicating that the preliminary efficacy of the intervention on self-efficacy varied depending on the child's age, with older children showing a greater increase in parents self-efficacy compared to their counterparts. The mean differences pre- and post-intervention within the interaction between parental self-efficacy and parent gender did not emerge as significant within the model.

#### Accelerometry sleep outcomes

**Figure 3:**
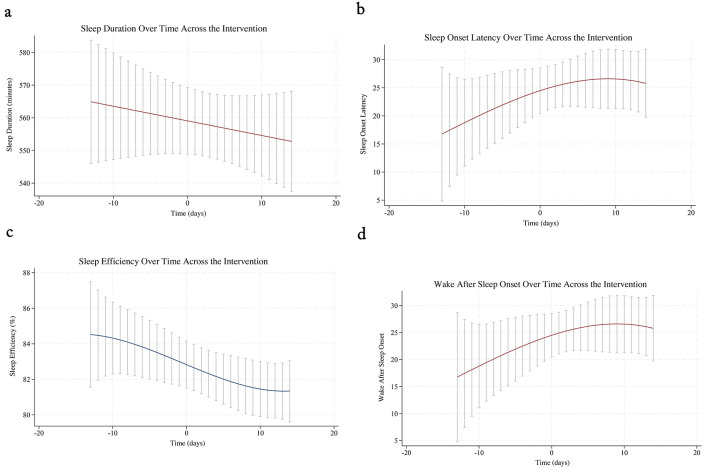
Changes in sleep outcomes across the intervention period **(a)** Sleep duration over time across the intervention; **(b)** Sleep onset latency over time across the intervention; **(c)** Sleep efficiency over time across the intervention; and **(d)** Wake after sleep onset over time across the intervention.

Sleep duration, SOL, and WASO did not significantly improve at the inflection point of intervention (*p*'s = 0.727, 0.868, and 0.092; See Figures 3a, b, d; See Table 4. Sleep efficiency significantly declined at the inflection point of intervention, indicating that the intervention may worsen sleep efficiency during the intervention delivery period (*p* < 0.001; See Figure 3c; See Table 4).

### Qualitative findings

#### Theme I: acceptability

The study was generally well-received by parents, who highlighted several aspects they found particularly helpful, especially in terms of the knowledge gained and the personalized interventions provided to help their children. A significant portion of parents (83.33%) reported that the knowledge they gained from the study was highly beneficial. They appreciated learning about sleep requirements and effective sleep strategies for their children. Parents also valued the specific, personalized interventions that were tailored to their children's needs; 88.89% of parents found these interventions helpful, as they provided concrete actions to address sleep issues. Parents also mentioned specific interventions that worked well, such as the bedtime pass and the worry time technique. The study's tools, such as the ActiWatch, were well-received by some parents, enhancing engagement and motivation. 44.44% of parents reported that their children enjoyed wearing the ActiWatch. Additionally, 22.22% of parents enjoyed tracking their child's sleep with the sleep diary. These findings suggest that increased engagement with sleep monitoring was a helpful component of the intervention. The general sentiment toward the study was positive, with 94.44% of parents finding the study helpful overall. However, parents in the study expressed some concerns regarding the acceptability of the intervention. Notably, 61.11% of parents reported that they desired additional interventions or content. The interaction with study staff also received mixed feedback. While some appreciated embedded text reminders regarding wearing the actigraphy device, and completing sleep diaries, 33.33% found the timing of texts inconvenient (see Table 5).

#### Theme II: feasibility

Parents generally found the study to be feasible and manageable, though a significant portion of parents (83.33%) noted some challenges of integrating the study into their daily lives (e.g., largely centering around balancing study requirements with their everyday activities, such as attending musicals, dealing with school breaks, and maintaining other routines). However, despite these challenges, the majority of participants (88.89%) reported that the study was easy to participate in and that they appreciated the straightforward design and requirements. This suggests that the study's design was accessible and manageable for most families (see Table 5).

#### Theme III: impact

The study had a varied impact on participants, with significant positive outcomes reported by 72% of parents, with 27.78% of participants attributed the positive outcomes toward specific sleep improvements resulting from the interventions provided during the study without prompt. Key themes that were commonly endorsed include increased awareness and knowledge about sleep (94%), improved sleep habits and behaviors (72%), and heightened motivation for better sleep across both children (56%) and parents (55%). A substantial number of parents (77.78%) reported that the study heightened their awareness of their child's sleep patterns. Additionally, almost all parents (94.44%) indicated that their knowledge about sleep improved through their involvement in the study. Furthermore, a significant portion of participants (72.22%) observed improvements in their child's sleep, including better sleep habits. However, 27% of the sample reported they did not find that their knowledge increased, as they believed they had a good baseline knowledge to begin with. Nearly all participants (94.44%) felt better equipped to help their child with sleep issues in the future (i.e., self-efficacy; see Table 5).

Without prompting, a third of the parents (33.33%) indicated that they planned to continue using the strategies they learned during the study, indicating a commitment to maintaining the improved sleep practices; additionally, a third of the participants (33.33%) reported an increased prioritization of sleep as a critical aspect of their child's health and development.

## Discussion

This multi-methods pilot study found that parental knowledge of healthy child sleep practices was limited, with only 43.7% of parents answering at least eight of ten knowledge items correctly. The intervention effectively increased parental sleep knowledge and led to moderate improvements in sleep health and large reductions in pediatric insomnia severity. We also observed a moderate increase in parents' self-efficacy, particularly among parents of older children. However, actigraphy-based measures such as sleep duration and SOL did not improve, and sleep efficiency worsened. Despite these findings, parents generally found the intervention helpful, acceptable, and feasible. These results demonstrate that such a brief behavioral intervention could be impactful in promoting healthy sleep practices in school-aged children and their parents, while also highlighting areas for refinement.

For our first aim, we found that parents were familiar with some sleep health basics (e.g., screen use, bedtime routines), but many did not know how much sleep their child needs or how to identify signs of adequate rest. These findings are consistent with past research showing parental confusion around sleep duration (Owens and Jones, 2011a; Owens et al., 2011b; Schreck and Richdale, 2011). Our community sample showed higher baseline knowledge than more urban or clinical samples, which may reflect its demographic makeup (e.g., white, educated, and higher income). Although we did not assess whether knowledge predicted sleep outcomes, existing literature suggests such knowledge is a robust predictor of sleep duration, quality, and habits (Owens and Jones, 2011a; Owens et al., 2011b; Schreck and Richdale, 2011). These findings highlight the importance of parent education in promoting healthier sleep in school-age children.

The qualitative findings from our second aim underscored high acceptability. Parents appreciated the personalized evidence-based practices and reported increased confidence in supporting their child's sleep. These findings align with the Theoretical Framework of Acceptability (TFA), which conceptualizes acceptability as multi-dimensional and includes domains such as affective attitude, burden, intervention coherence, perceived effectiveness, and self-efficacy (Sekhon et al., 2017, p. 8). In our interviews, parents' descriptions of the intervention as helpful and personalized map most closely onto perceived effectiveness and affective attitude, and reports of increased confidence implementing strategies are consistent with the self-efficacy domain. Additionally, comments regarding ease of participation and barriers to consistency reflect burden and opportunity costs. Parent's endorsement of practical barriers and scheduling demands suggest a need for flexible delivery models, such as group sessions or telehealth (Kwon et al., 2022). Even still, most parents reported gains in knowledge, motivation, and behavioral change. Several even noted their intention to continue sleep strategies beyond the study period. These studies, along with prior studies (Goodday et al., 2014), demonstrate that brief, parent-focused behavioral sleep interventions are both acceptable and impactful.

An important consideration for dissemination is the role of actigraphy within this intervention. Although actigraphy was used in the current study to support individualized feedback, promote sleep monitoring, and enhance parent engagement, it is not intended to be a core or required component of the intervention model. The primary mechanisms of intervention (including parent report, standardized questionnaires, motivational interviewing, and functional analysis) can be implemented without objective sleep monitoring. As such, this intervention could be delivered in clinical, school-based, or primary care settings where actigraphy is not readily available. Future implementation studies may examine whether actigraphy-enhanced feedback improves engagement or outcomes relative to parent-report and measurement-only delivery.

For the third aim, we demonstrated statistically significant improvements in parent-reported child sleep disturbance and child insomnia severity, with effect sizes consistent with prior studies in neurodivergent populations, clinical samples, and younger children (Byars and Simon, 2014; Pattison et al., 2020; Papadopoulos et al., 2019; Quach et al., 2011). Specifically, the mean CSHQ global scores decreased by ~2.2 points and the PISI scores decreased by ~2.1 points. While these changes were statistically reliable and associated with moderate to large effect sizes, they did not consistently reach thresholds typically considered clinically meaningful, particularly in a community-based sample in which most children fell below established clinical cutoffs at baseline. Indeed, the majority of children fell below established clinical thresholds on the CSHQ and had relatively low baseline PISI scores, suggesting that this sample is best characterized as consisting primarily of generally healthy sleepers with a smaller subset of children exhibiting elevated sleep disturbance.

Similarly, actigraphy data did not show improvements in duration (which decreased by ~8 min) or SOL (which increased by ~1 min), echoing prior research showing limited objective changes with brief interventions (Kidwell et al., 2019; Spaeth et al., 2024). One possible explanation is that behavioral sleep interventions may initially disrupt sleep timing or duration as families implement new routines, with improvements emerging over longer follow-up periods. For example, a participant who was working to make their wake time more regular day-to-day and weekend-to-weekend may initially be sleeping less as they implement that change. These findings reinforce the importance of combining subjective and objective data in future studies (Becker et al., 2022).

In this context, clinical significance is best interpreted relative to baseline symptom severity, functional impact, and parent-perceived benefit rather than statistical change alone. Qualitative interviews helped contextualize these findings, as parents rarely described dramatic or transformative changes in sleep parameters. Instead, parents emphasized increased awareness of sleep needs, improved bedtime routines, and greater confidence in managing sleep-related challenges, suggesting that perceived benefits aligned more closely with sleep health promotion and prevention than with the treatment of clinically severe sleep problems. Taken together, these findings suggest that while brief, personalized behavioral interventions may support sleep health promotion and prevention in community samples, their effects may be more pronounced among children with clinically significant sleep difficulties. Future adequately powered trials should intentionally recruit and stratify clinical and subclinical samples to directly test whether intervention effects differ as a function of baseline sleep disturbance severity.

For the fourth aim of the paper, we observed that the intervention significantly improved parental self-efficacy, especially among parents of older children. While research on sleep-specific parental self-efficacy in school-aged children is limited, infant sleep studies suggest a strong link between self-efficacy and positive sleep outcomes (Carroll et al., 2024; Cato et al., 2024). There have been few studies that have examined the effects of a brief behavioral sleep intervention on parent's self-efficacy, which generally find that self-efficacy is improved throughout intervention periods as parents learn and implement new skills (Brandhorst et al., 2016). Because parental self-efficacy is associated with improvements across various child health domains, including obesity and chronic illness management (Duraccio et al., 2021; Kieslinger et al., 2021), this finding highlights preliminary insights into the broader utility of brief, parent-focused interventions.

In our exploratory analyses, we observed (contrary to expectations) that sleep efficiency worsened during the intervention period. This may reflect short-term disruptions as families adjust to new sleep routines, a trend noted in other brief intervention studies (Hart et al., 2022; Kidwell et al., 2019; Paavonen et al., 2016). While we speculate that efficiency could increase with longer intervention implementation and follow-up, future RCT with longitudinal follow-up studies are needed to confirm this (Mitchell et al., 2019). Similarly, WASO did not improve, which aligns with previous findings suggesting that changes in night wakings may require longer intervention periods or emerge over time (Becker et al., 2022; Loring et al., 2016). Additionally, a ceiling effect may have limited detectable change in WASO, as even children with insomnia may not exhibit elevated WASO at baseline, reducing sensitivity to intervention-related improvements. These findings reinforce the importance of using both subjective and objective data to capture the full scope of intervention effects, particularly in community-based samples where subtle but meaningful changes may not be immediately evident in standardized measures.

This study fills a critical gap in pediatric sleep research by focusing on school-aged children, a population often overlooked (Meltzer et al., 2021). Strengths include the use of both qualitative and quantitative methods, multimodal sleep assessment, personalized interventions, and clinical oversight of the intervention delivery. The brief nature of the intervention makes it scalable and accessible. However, the has several limitations. Sample diversity was limited due to English-only intervention, self-selection bias, and overall individual homogeneity, as a pilot study, the small sample size restricted power to detect small to medium effects on primary outcomes. Measurement limitations included the absence of validated measures of parental self-efficacy related to the child's sleep, and the use of the Parental Knowledge questionnaire that is not yet validated for research purposes, potentially limiting construct validity. Further, on nights without diary-reported bedtimes, SOL was estimated using actigraphy-defined rest intervals, which may have reduced precision and contributed to null findings. Study design constraints also limited conclusions about the maintenance of gains over time due to not having further follow-up assessments, and while actigraphy enhanced feedback and engagement in this study, its use may limit dissemination in some settings. Lastly, Melatonin use was not directly measured in this sample, and the lack of a control group limited the ability to determine causality regarding the effectiveness of the sleep health promotion efforts. Given these limitations, future work should incorporate longer longitudinal follow-up assessment to determine whether treatments gains are maintained over time and conduct randomized controlled trials to better establish causality regarding intervention effectiveness. Future studies should also directly measure melatonin use to evaluate the role is plays in sleep health promotion efforts, particularly given evidence that melatonin may influence treatment outcomes in children with insomnia (Van Dyk et al., 2024). Additionally, recruiting more diverse samples will be critical to addressing limitations related to self-selection, language, and representation. Finally, although actigraphy enhanced feedback and engagement in the current study, future research should evaluate intervention delivery without actigraphy to optimize scalability and dissemination across settings.

In conclusion, these findings highlight the potential of brief, personalized behavioral interventions to increase parental knowledge, enhance self-efficacy, and improve sleep behaviors that reduce sleep disturbance in school-aged children. With high acceptability and feasibility ratings, this intervention model offers a promise in providing a practical, scalable strategy that pediatricians, psychologists, and schools can implement to expand access to sleep health services and support healthier sleep in children.

## Data Availability

The raw data supporting the conclusions of this article will be made available by the corresponding author upon reasonable request.
