# Supplementary material for: A multi-method pilot exploration of a brief behavioral sleep intervention for school-aged children: feasibility, acceptability, and initial evaluation
Source: Front Sleep. 2026 May 5;5:1731331. doi: 10.3389/frsle.2026.1731331 (PMC13183519; doi:10.3389/frsle.2026.1731331)
Supplement: Supplementary file 1 [file Table_1.docx]

**Supplemental Table 1**

*Accelerometry Measured Outcomes Model Fit*

| **Outcome** | **Linear Model Vs. Cubic Model** | | **Linear vs. Quadratic Model** | |
| --- | --- | --- | --- | --- |
|  | **LR chi-squared(df)** | ***p*** | **LR chi-squared(df)** | ***p*** |
| Sleep Duration | 7.75(2) | 0.093 | 0.040(1) | 0.527 |
| Sleep Efficiency | 0.45(2) | 0.799 | 0.24(1) | 0.622 |
| SOL | 1.48(2) | 0.478 | 1.45(1) | 0.228 |
| WASO | 1.13(2) | 0.567 | 0.02(1) | 0.880 |

*Note.* LR is an abbreviation for Likelihood Ratio.

**Supplemental Table 2***: Correlation Table Detailing Correlation Values between all Outcome and Potential Covariates*

|  | Child Mental Health | Child Sleep Habits | Pediatric Insomnia Severity | Parental Knowledge of Healthy Child Sleep | Parental Self-Efficacy | Baseline Average Sleep Duration | Baseline Average Sleep Efficiency | Baseline Average SOL | Baseline Average  WASO | Child Age | Parent Age | Income | Parent Highest Level of Education |
| --- | --- | --- | --- | --- | --- | --- | --- | --- | --- | --- | --- | --- | --- |
| Child Mental Health | 1.000 | .366* | .446** | -.109 | -.109 | .110 | -.374* | .109 | .007 | .060 | -.154 | -.018 | .460** |
| Child Sleep Habits | .366* | 1.000 | .621** | -.175* | -.133 | .070 | -.120 | -.059 | .199 | -.018 | -.093 | -.191 | .151 |
| Pediatric Insomnia Severity | .446** | .621** | 1.000 | .093 | -.012 | .079 | -.195 | .062 | .209 | .177 | . -.219 | -.013 | .201 |
| Parental Knowledge of Healthy Child Sleep | -.109 | -.175* | .093 | 1.000 | -.111 | .146 | .224 | -.152 | -.171 | .071 | .132 | -.030 | .091 |
| Parental Self-Efficacy | -.109 | -.133 | -.012 | -.111 | 1.000 | .227 | .098 | -.088 | .007 | .-370** | .042 | -.001 | -.251 |
| Baseline Average Sleep Duration | .110 | .070 | .079 | .146 | .227 | 1.000 | .184 | -.128 | .072 | -.139 | -.023 | -.037 | .025 |
| Baseline Average Sleep Efficiency | -.374* | -.120 | -.195 | .224 | .098 | .184 | 1.000 | -.327* | -.759** | .212 | .168 | -.107 | -.015 |
| Baseline Average SOL | .109 | -.059 | .062 | -.152 | -.088 | -.128 | -.327 | 1.000 | -.016 | .004 | -.019 | .042 | .121 |
| Baseline Average WASO | .007 | .199 | .209 | -.171 | .007 | .072 | -.759** | -.016 | 1.00 | -.207 | -.173 | .068 | .046 |
| Child Age | .060 | -.018 | .177 | .071 | .-370** | -.139 | .212 | .004 | -.207 | 1.000 | .098 | -.044 | .116 |
| Parent Age | -.154 | -.093 | -.219 | .132 | .042 | -.023 | .168 | -.019 | -.173 | .098 | 1.000 | .254 | .087 |
| Income | -.018 | -.191 | -.013 | -.030 | -.001 | -.037 | -.107 | .042 | .068 | -.044 | .254 | 1.000 | .046 |
| Parent Highest Level of Education | .460** | .151 | .201 | .091 | -.251 | .025 | -.015 | .121 | .046 | .116 | .087 | .046 | 1.000 |

** Correlation is significant at the 0.01 level (2-tailed)

 * Correlation is significant at the 0.05 level (2-tailed)
